# Family-based substance use screening and intervention for adolescents with chronic medical conditions: a study protocol to implement SBIRT-family within school-based health centers

**DOI:** 10.3389/frhs.2025.1469198

**Published:** 2025-06-24

**Authors:** Faith Summersett Williams, Natalie A. Larez, Lauren Mondesir, Kennedy Curtis, Sara Valdivia, Sara Becker, Kenneth Papineau, Aaron Hogue

**Affiliations:** ^1^ Division of Adolescent and Young Adult Medicine, Ann & Robert H. Lurie Children’s Hospital of Chicago, Chicago, IL, United States; ^2^ Department of Psychiatry and Behavioral Sciences, Ann & Robert H. Lurie Children’s Hospital of Chicago, Chicago, IL, United States; ^3^ Department of Medical Edcuation, Northwestern University Feinberg School of Medicine, Chicago, IL, United States; ^4^ Center for Dissemination and Implementation Science, Northwestern University Feinberg School of Medicine, Chicago, IL, United States; ^5^ Office of Student Health and Wellness, Chicago Public Schools, Chicago, IL, United States; ^6^ Partnership to End Addiction, New York, NY, United States

**Keywords:** adolescents, chronic medical condition, substance use, alcohol use, SBIRT, school based health centers (SBHC)

## Abstract

**Background:**

Adolescents with a chronic medical condition (CMC) have an increased risk of developing a substance use (SU) disorder, despite the impact that SU may have on disease-related outcomes. School-based health centers (SBHCs) offer universal screening, brief intervention, and referral for adolescents with chronic medical conditions for substance use treatment. Screening, Brief Intervention, and Referral to Treatment (SBIRT) is an evidence-based early intervention used to detect and address risky substance use that has yet to be broadly adopted in public schools. Moreover, despite extensive research supporting caregiver involvement in treatment for adolescent substance use, SBIRT models that actively engage caregivers are lacking. The primary goal of this qualitative study is the identification of contextual determinants (e.g., barriers and facilitators) of SBHCs implementation potential and adaptation needs of a family-based SBIRT protocol for integration into SBHCs.

**Methods:**

We are conducting this study in two SBHCs within the Chicago Public School system. In these SBHCS we are conducting focus groups with school partners (∼ 30 SBHC staff,∼25 adolescents with chronic medical conditions, and∼25 caregivers). Focus groups will be audio recorded and conducted in English. The semi-structured focus group guides were designed based on the Health Equity Implementation Framework (HEIF) and the Consolidated Framework for Implementation Research (CFIR). We will develop a codebook based on emerging codes from the transcripts and constructs from HEIF and CFIR. Emerging themes will be summarized highlighting similarities and differences between and within the different groups and SBHCs. Descriptive statistics and chi-square tests of associations will be used to assess the distribution of responses on the assessments between the different sites.

**Discussion:**

This study will describe key implementation determinants and SBIRT-Family adaptation needs from the perspective of multiple end-users. Results will provide insights for a randomized pilot hybrid type 2 effectiveness implementation study of the adapted SBIRT-Family model in two SBHCs assessing effectiveness outcomes (SU and linkage to treatment) and implementation outcomes (reach, adoption, equity, and cost). This research protocol will provide formative data to inform the development of a highly scalable approach that can be used in SBHCs across the country to serve a vulnerable population of adolescents with chronic medical conditions.

## Introduction

Adolescents with a chronic medical condition have been reported to be at increased risk for developing a substance use (SU) disorder. For instance, adolescents with chronic medical conditions are more likely to misuse alcohol, cannabis, and nicotine and initiate SU at younger ages compared to adolescents without chronic medical conditions (1–3). Importantly, engaging in SU may have an impact on their disease-related outcomes. SU in this population has implications for academic (engagement, grades, attendance), behavioral health (e.g., anxiety, depression, traumatic stress) and medical health outcomes (e.g., problematic medication interactions, treatment non-adherence, and poor disease management) (3).

In the United States, adolescents with chronic medical conditions such as asthma, diabetes, sickle cell, epilepsy, and cystic fibrosis, account for the bulk of pediatric ED visits and inpatient hospitalizations (4, 5). While inpatient hospitalizations provide acute medical management, many adolescents receive ongoing care in outpatient settings particularly in integrated primary care systems in community settings such as school-based health centers (SBHCs) (2). SBHCs are a nationally recognized model of care delivery that provide children, adolescents, and caregivers with accessible, comprehensive health services, directly within school environments. These centers are often staffed by interdisciplinary teams, including nurse practitioners, physicians, social workers, and behavioral health specialists, and offer services ranging from physical exams and immunizations to mental health counseling and chronic condition management (6). SBHCs exist across diverse educational settings in the United States and are especially common in schools serving historically underserved populations. Their purpose is to reduce barriers to care by eliminating logistical challenges such as transportation, missed school, or lack of parental availability. Nationally, more than 2,500 SBHCs serve nearly 6.3 million students annually, with funding and policy oversight varying by state (7).

In our specific study setting—a large urban SBHC network in the Midwest—SBHCs are embedded in schools and managed through collaborative partnerships between the school district and a mix of federally qualified health systems and hospital-affiliated health centers. Federally qualified health centers are community-based organizations that receive federal funding under Section 330 of the Public Health Service Act and are required to provide comprehensive care to medically underserved populations, often on a sliding scale (8). In contrast, hospital-affiliated health centers are operated by academic medical centers and offer integrated specialty care and have greater access to institutional resources, training, and research infrastructure (see Table 1).

**Table 1:** Comparison of SBHC models and local study context.

Model infrastructure components	FQHC-sponsored SBHCs	Hospital-affiliated SBHCs	Local study context
Funding	Federally funded	Institutional or hospital funding	Hospital-funded project in partnership with local school district and FQHCs
Target population	Medically underserved communities	Medically complex populations	Youth with chronic conditions
Governance	Community-based board	Hospital or academic oversight	Administered by hospital with collaboration from school district partners and FQHCs
Scope of services	Comprehensive	Comprehensive	Preventive care, chronic disease management, behavioral health
Integration with specialty care	Typically limited	High integration (e.g., shared EHRs)	Close collaboration with pediatric subspecialists
Staffing model	PCPs, behavioral health clinicians, RNs, MAs	PCPs, behavioral health clinicians, RNs, MAs	PCPs, behavioral health clinicians, RNs, MAs
SBIRT implementation status	Variable; often limited adoption	Not widely implemented or adapted for chronic conditions	Behavioral health screening in place; SBIRT not yet formally implemented or adapted

Many adolescents with chronic medical conditions are often prescribed pain medications to address pain control related to their conditions. Clinicians in pediatric treatment settings such as SBHCs are therefore faced with the challenge of appropriately addressing pain control, while preventing and addressing opioid misuse among adolescents with chronic medical conditions in the current climate of the opioid misuse epidemic (9). Clinicians in SBHCs also must be poised to address behavioral health concerns such as SU—behavioral health concerns are the focus of a large proportion of visits in SBHCs. Having access to SBHCs increases the likelihood of adolescents receiving services to address these concerns (10–12). Therefore, SBHCs offer a unique setting to address and prevent the escalation of SU in adolescents with chronic medical conditions (13).

Screening, Brief Intervention, and Referral to Treatment (SBIRT) for adolescents is an evidence-based approach to prevent and address risky SU (13, 14). The goal of the SBIRT model is to employ universal screening using a validated tool [e.g., Screening to Brief Intervention (S2BI)] (15, 16) to identify adolescents at risk of SU disorders, administer appropriate brief intervention such as motivational interviewing, and initiate referral to treatment (17, 18). SBIRT is widely endorsed as a universal strategy for detection and early intervention of SU and is recommended as a part of routine care in a variety of settings (13). Implementation of SBIRT in SBHCs has been associated with reductions in alcohol and cannabis use among adolescents but has not yet been widely adopted (6, 10, 11). SBHCs present a unique opportunity to adapt SBIRT for adolescents with chronic medical conditions to address modifiable SU risk, improve academic outcomes, and reduce related disease burden.

Standard SBIRT models, both within and beyond SBHCs, focus almost exclusively on the adolescent by including only the adolescent in the screening, intervention, and referral processes (12, 13). Yet, evidence indicates a need for major improvements in SBIRT engagement, effectiveness, and feasibility for adolescents in this setting (19–21). Involving caregivers (e.g., biological parents or legal guardians) could enhance the effectiveness of each phase of the SBIRT process by broadening the approach, as caregivers are vital sources of information regarding adolescent SU and mental health. During the screening phase, the use of multiple sources of information has been shown to be more accurate than relying on a single source for adolescent SU (22, 23). Proactively incorporating caregivers in the screening process for adolescent SU may be helpful to increase the likelihood of detection when youth have minimized self-report (24, 25). Pertaining to the BI phase, BIs for adolescent SU that incorporate parents have shown preliminary effectiveness (26, 27) and are likely to have considerable added value over adolescent-only BIs (28). Notably for this population, caregivers of adolescents with chronic medical conditions who have behavioral health concerns for their children often seek guidance from health clinicians (29). BIs can also help caregivers to learn effective parenting strategies (e.g., communication, coping, problem solving) that have been shown to reduce the risk of adolescent SU (30) and that are likely to be especially important for adolescents with chronic medical conditions who have special healthcare needs. Such caregiver strategies are likely to be especially important for adolescents with chronic medical conditions whose caregivers are likely to communicate with SBHC staff more extensively than caregivers of adolescents without chronic medical conditions. Regarding RT, influencing adolescents to enroll in SU services typically requires family involvement (28, 31). SBHC clinicians are well positioned to shape caregiver attitudes towards treatment options given their authority on healthcare issues (28, 29, 31).

Although SBIRT is widely endorsed, it is underutilized in SBHC settings, it has limited effectiveness, and questions remain about optimal SBIRT delivery strategies in SBHCs (17, 32, 33), particularly for adolescents with chronic medical conditions. Moreover, even though research strongly supports involving caregivers in treatment for adolescent SU (34–37), SBIRT models that actively engage caregivers are lacking. These limitations highlight the need for comprehensive evidence from school partners (e.g., SBHC staff, adolescents, and caregivers) on factors that will facilitate or impede the delivery and uptake of SBIRT in SBHCs for adolescents with chronic medical conditions. The primary goal of the current qualitative study is the identification of key contextual determinants (e.g., barriers and facilitators) to inform the selection of implementation strategies of a caregiver adapted SBIRT on substance use for adolescents with CMC receiving care in SBHCs.

This study is poised to advance research on SBIRT delivered in SBHCs in four primary ways. First, the standard SBIRT approach implemented in other state SBHCs focuses almost exclusively on the general adolescent population and not specifically on adolescents with chronic medical conditions. Second, conventional SBIRT targets adolescents only, without caregiver involvement, in screening and intervention procedures (13, 38). Third, while best practices for integration of SBIRT in SBHCs are emerging (38), implementation remains low nationally (13). Lastly, more research is needed to understand how family-focused SBIRT implementation determinants in SBHCs impact SU outcomes for adolescents with chronic medical conditions.

## Methods and analysis

This study is a part of a NIDA-funded multidisciplinary research network dedicated to promoting Family Involvement in Recovery Support and Treatment (FIRST) for youth with substance use problems (Grant number R24DA051946). As part of its mission, the FIRST Network provides expert feedback on study protocols that align with its scientific priorities. The protocol has not yet undergone formal peer review by a journal or other independent funding body.

Adolescents with chronic medical conditions, caregivers, and SBHC staff will be recruited from 2 SBHCs in Chicago, Illinois operated by Erie Health Services and UI Health. These two SBHC Clinic operators were selected because they have previously partnered in research conducted by Ann & Robert H. Lurie Children's Hospital, the children's hospital associated with the academic medical center where the Principal Investigator (PI, first author) is based. Erie Health Services and UI Health have a longstanding history of research collaboration with the PI's Department and Division. All study procedures and materials will be approved by the governing IRB.

### Study design

This study has two main aims. Aim 1: Collaborate with adolescents with chronic medical conditions and their caregivers to identify contextual determinants of SBIRT adaptation and implementation for engagement, cultural relevance, and usability using CFIR and HEIF. Aim 2: Engage with SBHC staff (nurses, physicians, techs, social workers, support staff, school administrators) to identify contextual determinants of SBIRT-Family adaptation and integration into clinic workflows using CFIR and HEIF.

Research objectives for Year 1 include: (1) evaluating contextual factors impacting potential SBIRT-Family implementation using qualitative approaches consisting of site visit observations and initial focus groups with adolescents, caregivers, and SBHC staff to refine focus group tools and guides for Year 2 focus groups. Year 2 involves (1) focus groups to obtain adolescent, caregiver, and SBHC staff input regarding adaptation of SBIRT-Family for adolescents with CMC. These findings will inform a future pilot hybrid type 2 effectiveness implementation study of the adapted SBIRT-Family model in two SBHCs assessing SBIRT-Family effectiveness outcomes (SU and linkage to treatment) and implementation outcomes (reach, adoption, equity, and cost). The study final design will be determined in collaboration with community partners following this formative phase.

The SBIRT-Family model that will be adapted for this population and setting is designed to optimize SBIRT developmental fit for adolescents by adding research-supported features based on research evidence (22, 36), developmental theory (25, 39), and best-practice pediatric care (12, 40). The model systematically includes caregivers in every SBIRT component: *Screening*: Adolescents complete an evidence-based screening tool for SU consumption and risk factors (41), while caregivers report concerns, they have about their child's SU and also co-occurring mental health problems (42). *Brief Intervention:* Adolescents receive digital education in the waiting area about SU prevalence rates and developmental neurobiology, followed by (depending on youth SU risk level) brief motivational interventions for SU reduction from providers during the clinical encounter (43); caregivers receive digital education in the waiting area about SU-preventive parenting practices, followed by (depending on family SU risk factors) brief motivational interventions for effective youth-caregiver communication about SU from providers during the clinical encounter (44); providers then convene a brief structured conversation between adolescents and caregivers to practice positive SU communication (45). *Referral to Treatment:* when clinically indicated, providers recommend SU counseling to adolescents and caregivers together and facilitate a first appointment for families who agree (22).

Identifying contextual determinants is essential for promoting the ultimate goal of designing and piloting a SBIRT-Family protocol with strong feasibility and acceptability features in SBHC settings for adolescents with chronic medical conditions (22). This study will use Consolidated Framework for Implementation Research 1.0 (CFIR 1.0) (46) and the Health Equity Implementation Framework (HEIF) (47) to guide focus groups with three stakeholder groups to identify promising additions and adaptations to SBIRT-Family that would enhance fit in the SBHC setting in CPS.

## Qualitative focus group guide development

We will use semi-structured focus group guides to identify and describe determinants of implementation of SBIRT-family in two SBHCs in Chicago. To explore the CFIR determinants, we will adapt the publicly available CFIR Universal Interview Guide to address the primary study questions. The CFIR guide has been widely used across a variety of populations and provides example qualitative interview questions based on CFIR determinants (Table 2) (48, 49). The interview will address all five CFIR domains: (1) intervention characteristics (e.g., compatibility of each element of SBIRT with usual care in the pediatric hospital setting), (2) partner characteristics (e.g., attitudes toward SBIRT, willingness to screen/be screened), (3) inner setting (e.g., compatibility of SBIRT with current medical services), (4) outer setting (e.g., policies and incentives to implement SBIRT, and (5) process (e.g., strategies to support SBIRT implementation) (Table 3) (50). To explore HEIF determinants, we will use the publicly available HEIF interview guide (Table 4) (47) to address domains known to affect health disparities and equity: (1) culturally relevant factors, such as medical mistrust, demographics, or biases of recipients (51–54); (2) clinical encounter or patient-provider interaction (55–57); and (3) societal context including physical structures, economies, and social and political forces (58–60).

**Table 2:** Assessments.

Concept	Measure	Description	Aim 1 students and caregivers	Aim 2 SBHC staff
Participant information	Demographics	Biological sex, race/ethnicity, age, gender identity, sexual orientation	X	X
Mental health	CRAFFT 2.1 + N	alcohol and substance use	X	
	Self-Stigma of Seeking Help Scale (SSOSH)	10 items; mental health treatment stigma	X	
	The Child and Youth Resilience Measure (CYRM-R)	17 items; protective factors	X	
SBIRT intervention evaluation	After-Scenario Questionnaire (ASQ)	3 items; Satisfaction	X	
	Client Satisfaction Questionnaire	Overall quality and expectations	X	
	Usability Testing	Post-Study System Usability Questionnaire (PSSUQ)	X	
SBIRT intervention implementation	HEIF	Focus group guide: patient-provider interaction, cultural factors of receipts, societal context	X	X
	CFIR 1.0	Focus group guide: Innovation, Outer Setting, Inner Setting, Individuals, Implementation Process,	X	X
	Organization Readiness for Implementing Change (ORIC)	12-items; commitment and readiness to implement change		X
	AIM	4-items; success for acceptability	X	X
	IAM	4-items; success for appropriateness	X	X
	FIM	4-items; success for feasibility	X	X

**Table 3:** Example SBIRT-family questions using CFIR.

CFIR domain	Question	Probe
Intervention characteristics	How does SBIRT-family compare to other similar existing substance use services in your SBHC?	What advantages does SBIRT have compared to existing programs?
		What disadvantages does SBIRT have compared to existing programs?
Inner setting	What kinds of infrastructure changes would be needed to accommodate SBIRT-family?	Changes in scope of practice?
		Changes in formal policies?
		Changes in information systems or electronic records systems?
Outer setting	How well do you think this drug and alcohol program would meet the needs of students with chronic medical conditions and families receiving healthcare at SBHCs?	How important is drug and alcohol use in comparison to other needs they may have?
		In what ways will this program meet their needs (e.g., improved access to services? Reduced wait times? Help with self-management? Reduced travel time and expenses)?
Individual characteristics	How do you feel about using SBIRT-family in your setting?	Do you have any feelings of anticipation? Stress? Enthusiasm? Why?
Process	Is a patient portal used at SBHCs?	If so, what is the name?
		Are students and parents familiar with this patient portal?
		Do they use the portal?

**Table 4:** Example SBIRT-family focus group questions using the HEIF.

HEIF Domain	Question	Probe
Clinical encounter (patient-provider interaction)	How do your conversations about your health and treatment plan usually go with your SBHC provider?	Do you feel not understood by your doctor or nurse?
		Have you ever felt treated differently from others when getting treatment for [health problem]?
Cultural factors of recipients	Do teens and parents trust SBHCs?	Do teens trust the healthcare providers at SBHCs?
		Do healthcare providers respect teens’ thoughts and concerns about their treatment plans?
Societal context	Do you feel there is a stigma against people, especially teens, who need help with drug and alcohol use?	Do you think these teens may be treated differently when getting help for drug and alcohol use?

## Focus group delivery

Separate student, caregiver, and SBHC staff focus groups will be conducted in person by a trained PhD-level behavioral scientist, last approximately 90 minutes, and use audio and graphic recording (i.e., translating the main themes and ideas discussed during the focus groups into a drawing). Adolescent patient and caregiver focus groups will be conducted by the principal investigator in groups of approximately 6–8 attendees. Audio recordings will be transcribed verbatim and graphic recording will be done in real-time. Graphic recording engages participants in real-time member-checking to validate understanding of responses. Participants will be asked to comment on or suggest corrections to the themes reflected in the graphic. The final drawing will be shared as a PNG file with participants via separate emails to preserve confidentiality. The drawing will exclude identifiers or information that reveal participants in the group. Data collection will stop or extend until data saturation is reached. Immediately following each focus group, the interviewer and/or facilitators will write corresponding field notes indicating contextual details and nonverbal cues. Undergraduate-level research assistants will review 5 minutes of all audio transcripts for accuracy. We will not return transcripts to participants for review. SBHC staff, students, and caregivers will receive food during the focus groups and $25 for their time and effort.

## Qualitative analysis

The transcribed and de-identified SBHC staff, student, and caregiver focus groups will be analyzed for thematic content (61) using a deductive approach to identify which CFIR and HEIF framework determinants influence SBIRT-family adaptation and program implementation (62). The research team will develop a codebook by pulling determinants directly from CFIR and HEIF and including code definitions and application guidelines. Two trained coders with qualitative experience will independently review and rate each transcript using a modified RADaR method (63). This method is acceptable for inductive and deductive analysis and has multiple advantages over qualitative software. Since our project is deductive, focused on identifying the relevance of identified facilitators and barriers to SBIRT implementation, this method allows us to spend less time coding and more time identifying, expanding, and linking themes. The RADaR method retains methodological rigor while reducing the costs associated with software training and burden. Using this method, the content from each transcript will be organized into a data table, codes applied, and inter-rater reliability assessed after the first two transcripts are coded. Upon completion, the coders will identify larger themes and corresponding subthemes to organize as a “thematic map” to illustrate potential thematic relationships. The two coders will then review the validity of and refine the thematic map by comparing it to interview transcripts. Once the thematic map is internally consistent and sufficiently describes the data, we will finalize the map's major and minor themes and provide corresponding participant quotations that depict determinants of SBIRT implementation.

During the coding process, the coders will meet weekly and consult with team members with expertise in implementation science and SBIRT to identify themes and constructs to refine the thematic map. After the thematic map is finalized, the coders will enter the CFIR determinants into the “CFIR-Expert Recommendations for Implementation” matching tool. The tool generates specific implementation strategies as potential candidates to implement SBIRT in the pediatric hospital setting, which will lay the groundwork for future work (64).

## Recruitment procedures

### Focus group participants

Study participants will be recruited through a combination of purposive and convenience sampling based on receiving or providing treatment at a SBHC for a CMC. To overcome potential challenges with recruitment the research team will use several strategies to support recruitment and engagement, including: (1) flyers posted in SBHCs and social media sites, (2) SBHC staff introducing the study to eligible participants, (3) emphasize confidentiality and voluntary participation, and (4) flexible consenting and engagement options (e.g., virtual). Interested participants will contact research staff for more information, and then be provided a link to a secure online questionnaire (administered via REDCap) to provide contact information. Research assistants will contact interested participants to schedule a meeting to assess inclusion criteria (detailed below). Caregivers will provide consent for adolescent participants and underage participants will provide adolescent assent. Caregivers, SBHC staff, and students 18 years old will provide consent for their own participation. Participants from two SBHCs with different clinic operators will be recruited to support the generalizability of the findings for a future pilot trial. All focus group participants will be provided a $25 gift card.

Aim 1: Collaborate with adolescents with chronic medical conditions and their caregivers to identify contextual determinants of SBIRT-Family implementation for engagement, cultural relevance, and usability using CFIR and HEIF. Drawing on CFIR and HEIF, we will generate formative data to establish: (1) individual barriers (e.g., stigma) and facilitators (e.g., trust in SBHC staff or perceived confidentiality of services) for SBIRT-Family engagement and (2) which ethical concerns (e.g., confidentiality) and cultural and contextual adaptations (e.g., inclusive language) are most relevant to addressing barriers to engagement. Usability and usefulness of the intervention will also be assessed.

### Aim 1 procedures

In Phase 1 of the focus groups, adolescents with chronic medical conditions (ages 14–18; *n* = 5) with an established medical treatment history at the identified SBHC in Chicago, Illinois, and caregivers (*n* = 5) will be recruited to participate in two separate focus groups developed through CFIR and the HEIF to assist with the refinement of the focus group tools. Due to a long-standing history of disparities in SU treatment and systemic inequities, adolescents with chronic medical conditions living in disinvested areas are likely have significant concerns related to being asked questions about SU in SBHCs. Thus, focus group facilitators will ask questions from the HEIF about perceptions of SU among adolescents with chronic medical conditions and barriers (e.g., confidentiality, stigma, and discipline policies) and facilitators (e.g., peer support and knowledge of treatment options) to SU treatment engagement.

Once feedback from the initial adolescent focus group is incorporated into the HEIF focus group guide, adolescents with chronic medical conditions (ages 14–18; *n* = 10) and caregivers (*n* = 10) from two SBHCs will be recruited to participate in Phase 2 focus groups, with two separate group meetings for each group to evaluate the SBIRT-Family prototype procedures. Focus groups will be conducted at two SBHCs with no more than eight participants per group.

Phase 2 focus group facilitators will also ask adolescents and caregivers to complete a mock interaction with the standard SBIRT-Family intervention (e.g., complete the CRAFFT 2.1 + N screener, BI and RT with one of the session facilitators). Adolescents will be informed of patient rights and confidentiality and that during this formative qualitative study, screening results will not be shared with caregivers. Interactions will be timed, audio and graphically recorded, and observed by two session facilitators. Following the completion of mock interactions, focus group participants will be queried about (1) their top ethical concerns about SBIRT-Family and its integration into SBHC clinics; (2) potential cultural adaptations that would further promote their engagement (e.g., inclusivity, jargon-free language); (3) feedback and recommendations for adaptations to the BI for youth with chronic medical conditions and caregivers (e.g., health information related to chronic illness and SU); and (4) functionality of the RT process for SU-specific treatment and mentoring-based organizations within their neighborhood. Specific queries about how adolescents would address such issues will be included. Participants will also be queried about what engaged their participation in the SBIRT-Family intervention and their preferred format for BI participation (e.g., information distributed in person vs. virtual; group format vs. individual). This will promote our ability to build upon existing engagement strengths. Usability and usefulness of the intervention will be evaluated through qualitative report, completion of self-report measures, and time to complete the BI and RT (see Aim 1 Assessments in Table 2).

### Aim 1 analysis

Audio recordings of the focus group sessions will be transcribed verbatim and uploaded into Dedoose, a qualitative data analysis platform that supports mixed-methods research. Qualitative data from the focus groups and quantitative data from self-report measures will be analyzed using a convergent mixed-methods design. Constructs from CFIR and HEIF will guide qualitative coding and interpretation. An initial codebook will be developed through a combination of deductive codes derived from CFIR and HEIF domains and inductive codes emerging from the transcripts and focus group guides. After iterative discussions and pilot coding to ensure consistency and consensus, the full dataset will be double-coded by trained team members. Interrater reliability will be assessed using pooled Cohen's kappa, and discrepancies will be resolved through team discussion. Quantitative survey data (e.g., demographic characteristics) will be analyzed using descriptive statistics to provide contextual information about the focus group participants. Frequencies, means, and standard deviations will be reported, and data will be stratified by partner group (e.g., adolescents, caregivers). Following separate analysis of qualitative and quantitative datasets, findings will be integrated using a joint display matrix to identify areas of convergence, complementarity, or divergence across data sources. This approach will allow us to compare and contrast themes across participant groups and assess how survey responses align or diverge from qualitative perspectives. Emerging themes that fall outside CFIR and HEIF constructs will be documented as new insights and may inform updates to existing implementation frameworks.

Aim 2: Engage with SBHC Staff to Identify Contextual Determinants of SBIRT-Family integration into clinic workflows using CFIR and HEIF.

SBHC staff recruited for focus groups will complete organizational assessments (e.g., observational studies of clinic workflows). The PI will clarify organizational readiness for implementing SBIRT-Family procedures and identify optimal workflows to engage adolescent patients around and within SBHC clinic appointments.

### Aim 2 procedure

Phase 1 focus groups will include SBHC staff [e.g., administrators, teachers, and clinicians (e.g., nurses, techs, and physicians; *n* = 5)] from one SBHC. Focus group facilitators will use CFIR and HEIF focus group guides to assist with the refinement of focus group tools.

Phase 2 SBHC staff will be recruited from two SBHCs, one operated by UI Health and one operated by Erie Health Services (*n* = 16), to participate in two separate focus groups evaluating prototype SBIRT-Family procedures and clinical workflow. First, focus group facilitators will lead SBHC staff through the refined CFIR and HEIF focus group guide to identify their needs, wishes, and anticipated barriers to SBIRT-Family implementation. Focus group participants will be probed to consider the implementation feasibility of the assessment protocol (i.e., the level of ease with which they could deliver or complete SBIRT-Family), how frequently SBIRT-Family should be administered to establish a SU treatment guideline, and preferred procedures for using SBIRT-Family. Finally, the focus group guide will include questions for SBHC staff regarding treatment workflow and electronic medical record (EMR) documentation procedures to inform SBIRT-Family protocol adaptation.

Second, observational studies of SBHC clinic workflows for primary care appointments with adolescents with CMC will be conducted across multiple times of day and days of the week (to cover possible variations in SBHC workflows across time). Observations of workflows and daily clinical procedures, which entail a researcher shadowing staff (e.g., noting the order and time required for typical activities), may help to identify issues that may not arise in focus groups with SBHC staff. The research team will maintain written (daily procedures, staff meetings) and audio-recorded (informal interviews) data logs to capture information gained from the site visits.

Third, co-design workshops will occur with SBHC staff to iteratively inform an implementation plan specific to their clinic; these will include cognitive walkthroughs of the tasks detailed in the plan to identify potential flaws. Iterations of the plan will progress until major flaws have been identified and addressed. Self-report assessments related to SBIRT-Family acceptability, appropriateness and feasibility will be administered and housed via a secure online platform (REDCap; Table 2).

Lastly, the research team and SBHC partners will use the Consolidated Framework for Implementation Research–Expert Recommendations for Implementing Change (CFIR-ERIC) matching tool to guide the systematic identification, selection, and tailoring of implementation strategies that align with the implementation determinants identified through the focus groups (65). Using the focus group data, the research team will map the identified determinants to the corresponding CFIR constructs (e.g., compatibility, relative advantage, available resources, leadership engagement) across domains such as inner setting, outer setting, and characteristics of individuals. Following this mapping process, the research team and SBHC partners will enter the prioritized CFIR and HEIF constructs into the CFIR-ERIC online matching tool, which cross-references each construct with a comprehensive list of expert-recommended implementation strategies. The tool will generate a ranked list of strategies with varying levels of endorsement based on prior expert consensus (e.g., identifying and preparing site champions, adapting and tailoring the intervention, revising professional roles, or developing educational materials). The research team will work collaboratively with SBHC staff to review the list of recommended strategies and select those that are both feasible within the SBHC context and aligned with staff-identified needs and existing workflows. Selected strategies will then be adapted and operationalized in preparation for a future multi-site hybrid trial. This iterative and participatory approach will ensure that the implementation plan for SBIRT-Family reflects both implementation science best practices and the practical realities of delivering integrated behavioral health services in SBHC settings.

### Aim 2 analysis

Mixed-methods analyses will be conducted using data collected from qualitative (e.g., workflow observations, co-design workshops) and quantitative sources (e.g., observation duration, feasibility, and acceptability ratings). A convergent parallel design will be employed to synthesize qualitative and quantitative data concurrently.

Quantitative data, including self-report scores from the Acceptability of Intervention Measure (AIM), Intervention Appropriateness Measure (IAM), and Feasibility of Intervention Measure (FIM), will be analyzed using descriptive statistics (means, SDs, and item-level distributions) (see Table 2). These data will help quantify SBHC staff's perceptions of the caregiver-adapted SBIRT model and highlight preliminary feasibility considerations. While these measures are widely used and validated in implementation research, they are known to exhibit ceiling effects, particularly in early-stage acceptability testing which may limit variability and sensitivity to subtle differences in staff perspectives. To address this limitation, item-level response distributions will be examined alongside means and standard deviations to detect any skewness or clustering of responses. Quantitative data from the workflow observations will be summarized using frequency counts and average durations for key tasks to inform the logistical feasibility of implementation.

Qualitative data from observational field notes and co-design sessions will be analyzed using thematic analysis, guided by CFIR and HEIF. Community partner suggestions regarding implementation processes, potential barriers, and delivery preferences will be coded and synthesized. As in Aim 1, deductive and inductive approaches will be used to capture both framework-aligned and new insights.

Following individual analyses, qualitative and quantitative findings will be compared and organized into a joint display matrix. This will facilitate integration of SBHC staff's narrative perspectives with numeric ratings and observational data. Integrated findings will be used to develop an initial implementation plan, including a short list of candidate implementation strategies tailored to the SBHC context. These strategies will inform the design of a future multi-site hybrid trial.

## Confidentiality and data storage

Focus group data will not be collected with identifiers and therefore will be less likely to be subject to breach of confidentiality. Any inadvertent disclosure of identity during focus groups will be removed from transcriptions. Audio data will be destroyed when the study is closed with the IRB. Focus group data, including visit checklists, audio files and transcriptions will be labeled with a study-ID specific to each site and participant group (e.g., Erie Health/UI Health—Adolescent/Caregiver/SBHC Staff) and stored securely. All study databases will be protected with password access systems and all datasets including focus group transcripts and questionnaires will be stores in a password-protected folder on a secure server managed by the study team. Transcripts will be entered into qualitative data management software for storage and analysis.

We have developed systematic protocols for data handling and storage. We maintain both paper files and computer files for each participant. Study records that contain names or other personal identifiers, such as informed consent forms, will be maintained separately and securely with limited access. Study questionnaires and any other forms that link participant numbers to identifying information will be secured with limited access. Paper/electronic files included are: (1) informed consent, (2) any paper or electronic forms (e.g., visit checklist, session notes), and (3) study assessments/questionnaires. These files have identifying information and are linked to the data by the patient identification number (PID). To protect the integrity of the participant's data study staff will assign each individual a unique PID at study enrollment. This code number will be used for all study data. We will maintain a list of participants with links between identifying information and code numbers for the sole purpose of avoiding any duplication in completion data collection sessions. Only study staff will have access to these lists, which will be kept behind double-locks or on a secure server with password protected access. Consents are stored electronically on a secure sever with password protected access and are only accessed in the event of a consent amendment or an audit.

The study questionnaires will be completed privately in REDCap, minimizing the risk of breach of confidentiality. The main study site will provide a standards-compliant (HIPAA, HHS Cybersecure) private cloud server for the hosting of application content in REDCap and user data for the duration of the application deployment. This is a secure system and will be further protected by login credentials for limited access, to protect participant confidentiality. Because focus group and questionnaire topics may cause psychological discomfort, we will remind participants that they may choose not to answer any questions that they do not wish to answer or choose to take a break from taking the assessment at any time.

Data files are exported from the computer software program and imported into SPSS database for storage and analysis. Computer data files never have any identifying information. Data files do not include information that could be used to identify the participant from the data file alone.

## Discussion

Adolescents with chronic medical conditions have high rates of risky drinking and drug use. Yet, they are not routinely screened for SU in SBHCs even though they frequently interact with the healthcare system for management of their chronic medical conditions, highlighting a major missed opportunity. This research protocol will facilitate the implementation of family-focused SBIRT procedures in SBHCs within the third largest public-school system in the country (66).

Implementation of an evidence-based substance use approach in SBHCs for adolescents with chronic medical conditions requires a thorough understanding of context-specific bottlenecks, factors that contribute to workflow integration, and predictors of SBHC staff adoption to protect as many adolescents with chronic medical conditions as possible from risky alcohol use and substance use that can lead to future dependence, overdose, or accidental death. Findings from this study will generate insights on implementation determinants that affect SBIRT-family implementation in SBHCs from the perspective of a diverse group of community members and SBHC staff at different levels of the organization in Chicago Public Schools. A variety of factors between and within settings and other key characteristics of participants (race and ethnicity, gender identity, location, role in the community, etc.) is expected to be prominent (67–70). In addition, the acceptability of the adapted intervention and the implementation plan may vary depending on participants’ awareness of existing evidence of brief family-focused substance use interventions (71, 72) and their status within the SBHC system. Given recent CPS initiatives to integrate substance use prevention and intervention into their programming, we do not expect measures of acceptability, appropriateness, and feasibility to vary among SBHC staff based on demographic characteristics.

This study will provide preliminary data to assess SBHCs’ readiness to implement family-focused SBIRT procedures and inform the development of a highly scalable approach that can help prepare the path for later feasibility testing in a fully powered multi-site randomized trial throughout CPS. Additionally, these findings will contribute to the development of setting-specific outreach, educational and training materials to disseminate evidence among different community members, all of which will contribute to increasing the health knowledge about alcohol and drug use among this vulnerable population of adolescents.

## Dissemination policy

The study team is committed to public dissemination of results of the formative research to participants, local community members and policy makers in Chicago, and the adolescent addictions scientific community. Dissemination of study results will follow principles of good participatory practice. Results will be published in conference abstracts and peer-reviewed journals. Study results will be disseminated through presentations to local community members and policymakers in Chicago, including Chicago Public Schools.
